# UV-Cured Methyl Methacrylate Electrolyte Prepared by Ultrasonic Spray Deposition

**DOI:** 10.1021/acsomega.6c03204

**Published:** 2026-05-26

**Authors:** Chi-Ping Li, Chia-Chi Chin

**Affiliations:** Department of Chemical Engineering, 59215 National United University, No. 2, Lienda, 360302 Miaoli, Taiwan

## Abstract

In this study, gel polymer electrolytes (GPE) containing lithium salts were fabricated using an ultrasonic spray deposition (USD) technique. Methyl methacrylate (MMA) was dissolved in propylene carbonate (PC) as the polymer precursor, with IRGACURE-184 employed as the photoinitiator and phenothiazine as the stabilizer. Lithium perchlorate (LiClO_4_) and lithium chloride (LiCl) were introduced as lithium-ion sources, separately. The precursor solutions were stirred at 60 °C for 12 h prior to film deposition to ensure homogeneity. The structural and electrochemical properties of the resulting electrolyte films were systematically characterized using UV–Vis–NIR spectroscopy, electrochemical impedance spectroscopy (EIS), optical microscopy, and scanning electron microscopy (SEM). The effects of processing parameters, including lithium salt type, lithium salt concentration, spray flow rate, and number of spray coats, on the optical transmittance and ionic conductivity were investigated. The results demonstrate that ultrasonic spray deposition enables the fabrication of uniform electrolyte films with high material utilization and scalability, making it suitable for low-dose thin-film deposition. The maximum optical transmittance values of the electrolyte films reached 60% for LiClO_4_ and 65% for LiCl, respectively. In terms of electrochemical performance, the ionic conductivity of LiClO_4_-based electrolyte films reached 1.075 × 10^–7^ S cm^–1^, which is significantly higher than that of LiCl-based films (6.151 × 10^–7^ S cm^–1^). Furthermore, mechanical characterization revealed that both LiClO_4_ and LiCl electrolyte films exhibited hardness values exceeding 1.6 GPa and elastic moduli greater than 2.8 GPa, indicating good mechanical stability. These results suggest that the developed gel polymer electrolyte films prepared by ultrasonic spray coating exhibit promising properties for applications in electrochemical and electrochromic devices.

## Graphical abstract

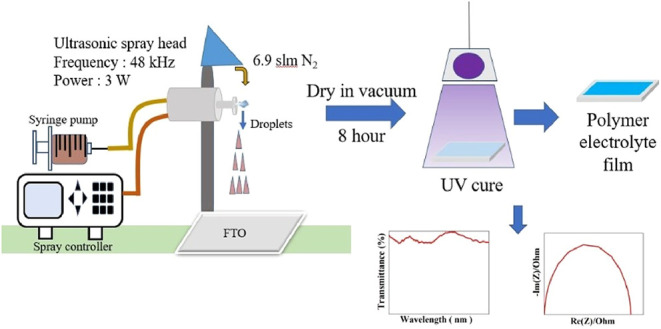

## 1 Introduction

The increasing demand for fossil fuel-based energy has led to the depletion of natural resources and has significantly contributed to global warming and climate change.^1,2^ As a result, considerable efforts have been devoted to the development of alternative energy sources and strategies for energy conservation and carbon reduction. Renewable energy technologies, including solar, wind, and geothermal energy, have been extensively explored.^3–5^ However, the intermittent nature of these energy sources limits their stability and reliability, thereby necessitating the development of efficient energy-storage systems such as rechargeable batteries.^6–11^

Electrolytes are key components in electrochemical devices and can be broadly classified into liquid electrolytes, solid electrolytes, and gel polymer electrolytes (GPE). Liquid electrolytes exhibit high ionic conductivity and are easy to process but suffer from leakage and safety concerns.^12–14^ Solid electrolytes provide improved safety and mechanical stability; however, their ionic conductivity is generally limited.^15,16^ Gel polymer electrolytes (GPE) combine the advantages of both systems and have attracted increasing attention due to their balanced properties, including enhanced ionic conductivity, improved mechanical strength, and reduced leakage risk.^17–20^

Ultrasonic spray coating has emerged as an effective technique for the fabrication of thin-film materials. This method utilizes high-frequency vibrations to atomize precursor solutions into fine droplets, enabling uniform deposition onto substrates. Compared with conventional coating techniques, ultrasonic spray coating offers advantages such as reduced material consumption, low processing cost, scalability for large-area fabrication, and precise control over film thickness and morphology.^21–27^ This technique has been successfully applied in electrochromic devices, including smart windows,^28–34^ supercapacitors,^35,36^ antiglare mirrors,^37–41^ and flexible electronic systems such as electronic skin.^42–46^

Poly­(methyl methacrylate) (PMMA) has been widely investigated as a host material for polymer electrolytes due to its excellent optical transparency, mechanical robustness, and chemical stability. PMMA-based electrolytes can achieve favorable ionic conductivity when combined with appropriate lithium salts and are typically fabricated via in situ polymerization or UV-induced cross-linking processes.^47–55^ Several studies have demonstrated the effectiveness of PMMA-based gel polymer electrolytes. For instance, Primiceri et al. reported an electrolyte system with an ionic conductivity of 1.9 × 10^–8^ S cm^–1^.^56^ Xu et al. achieved a significantly higher conductivity of 2.3 × 10^–3^ S cm^–1^ through UV cross-linking.^57^ Lu et al. demonstrated excellent electrochromic performance, including high optical modulation, fast switching response, and long-term stability.^58^ Additionally, Guan et al. reported an MMA–HEA–PEG-based electrolyte system using propylene carbonate (PC) as solvent, LiClO_4_ as lithium salt, and IRGACURE 184 as photoinitiator, exhibited a conductivity of 5.23 × 10^–6^ S cm^–1^.^59^

Despite these advances, the simultaneous achievement of high optical transparency, uniform film morphology, and controllable ionic conductivity in polymer electrolyte films remains challenging, particularly when scalable fabrication techniques are required. In addition, although ultrasonic spray coating has shown promise for thin-film deposition, its application in the fabrication of gel polymer electrolytes and the systematic investigation of processing parameters on electrolyte performance have not been fully explored.

In this study, methyl methacrylate (MMA) was employed as the polymer matrix with propylene carbonate (PC) serving as the plasticizer. IRGACURE 184 and phenothiazine were used as the photoinitiator and stabilizer, respectively. Lithium perchlorate (LiClO_4_) and lithium chloride (LiCl) were introduced as lithium-ion sources separately. Gel polymer electrolyte films were fabricated via ultrasonic spray coating, followed by UV curing. The effects of the lithium salt type, concentration, spray flow rate, and spray layer number on the optical transmittance and ionic conductivity were systematically investigated. The results demonstrate that the proposed approach enables the fabrication of uniform, transparent, and electrochemically functional electrolyte films, providing a promising strategy for applications in electrochemical and electrochromic devices.

This work provides new insights into the design and scalable fabrication of high-performance polymer electrolyte films and contributes to the development of next-generation electrochromic and energy-related devices.

## 2 Experimental Section

### 2.1 Materials

Methyl methacrylate (MMA, 99%, Thermoscientific), propylene carbonate (PC, 99%, Sigma-Aldrich), lithium perchlorate (LiClO_4_, 99%, Sigma-Aldrich), lithium chloride (LiCl, 99%, Sigma-Aldrich), IRGACURE 184 (99%, Sigma-Aldrich), phenothiazine (99%, Sigma-Aldrich), flourine doped tin oxide (FTO, TEC-15, Pilkington, 20/□) coated glass.

### 2.2 Preparation of Polymer Electrolytes Precursor Solution

Inside a glovebox, a lithium salt (LiClO_4_ or LiCl, at 0.1, 0.2, 0.3, 0.4 M) was added to a mixture of methyl methacrylate (MMA, 6 mL) and propylene carbonate (PC, 5 mL). Subsequently, IRGACURE 184 (0.55 g) and phenothiazine (0.44 g) were introduced into the solution. The resulting mixture was heated to 60 °C and stirred continuously for 12 h to obtain a homogeneous precursor solution.

### 2.3 Ultrasonic Spray Deposition (USD) of Polymer Electrolyte Films

The precursor solution was transferred into a syringe and equipped on a syringe pump (YSC SP series). A desktop automatic ultrasonic sprayer (DISPENSER TECH) equipped with an ultrasonic spray head (Sono-Tek) was used for film deposition. The spraying parameters were fixed at an operating frequency of 48 kHz and a power of 3 W. Nitrogen was used as the carrier gas with a flow rate of 6.9 standard liters per minute (slm). FTO glass substrates were used as the substrates. The spraying area was set to 25 mm × 18 mm of each FTO, with a nozzle moving speed of 30 mm s^–1^. Electrolyte films were deposited onto the FTO substrates by applying different spray flow rate (0.25 and 0.5 mL min^–1^) and numbers of spray coats (10 and 20 coats), respectively.

### 2.4 Curing Process

After deposition, the coated films were subjected to vacuum treatment for 8 h and subsequently exposed to ultraviolet (UV) irradiation (UVP, UVGL-58) at a wavelength of 365 nm for 10 min to cure the electrolyte.

### 2.5 Transmittance Measurement

The spray-cured electrolyte film was assembled into a sandwich structure by placing it face-to-face with another piece of conductive glass that had been precleaned with acetone and ethanol. The periphery of the assembly was sealed by using a hot-melt adhesive to form an enclosed cell, as illustrated in Figure 1.

**Figure 1:**
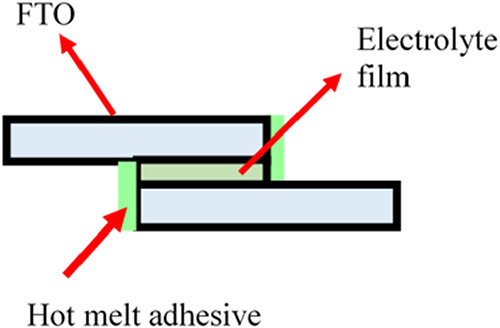
Assembled device for transmittance and electrochemical impedance spectroscopy (EIS) measurements.

The optical transmittance of the fully cured electrolyte films was measured using an optoelectronic spectroscopic system (Bio-Logic SEMSO) over a wavelength range of 400–800 nm in the visible region. Prior to measurement, two clean, uncoated FTO conductive glass substrate was used as the reference. The optical transmittance of the polymer electrolyte films was then evaluated at a wavelength of 550 nm. The surface morphology of deposited films was investigated by using an optical microscope (KEYENCE, VHX-S650E).

### 2.6 Impedance Measurement

EIS measurements were carried out using a potentiostat/impedance analyzer (Bio-Logic SP-150). The measurements were performed over a frequency range of 1–10^6^ Hz with an AC amplitude of 100 mV. The film thickness of the films was examined using a SEM (JEOL, JSM-5600).

The ionic conductivity (σ, S cm^–1^) of the electrolyte films was calculated according to Ohm’s law using the following eq 1

1 σ=L/AΩ

where *L* (cm) is the thickness of the electrolyte film, *A* (cm^2^) is the contact area, and *R* (Ω) is the bulk resistance obtained from the impedance measurements.

### 2.7 Hardness and Elastic Modulus Measurement

The mechanical properties of deposited films, including the hardness and elastic modulus, were measured using a nanoindenter (Hysitron, TI 980 TriboIndenter).

## 3 Results and Discussion

### 3.1 Optical Transmittance

Optical transmittance and ionic conductivity are key performance indicators for polymer electrolytes. The optical properties of the electrolyte films were evaluated using UV–vis–NIR spectroscopy under different spray flow rates (10 and 20 mL min^–1^) and coating layers (10 and 20 layers). The film thickness was measured by Scanning Electron Microscopy (SEM, JEOL JSM-5600). As shown in Figure 2 and Table 1, the transmittance of 0.1 M LiClO_4_ electrolyte films in the visible region (at 550 nm) ranged from 50 to 60%. It was observed that the number of coating layers had a more pronounced effect on transmittance than the spray flow rate. For the 0.2 M LiClO_4_ electrolyte, the transmittance remained relatively stable at approximately 60% under the same processing conditions, indicating that a moderate increase in salt concentration does not significantly affect optical transparency. However, when the concentration was increased to 0.3 M, the transmittance decreased to 46–58%. A further increase to 0.4 M resulted in an additional reduction in transmittance, ranging from 40 to 55%. The decrease in transmittance at higher lithium salt concentrations is attributed to the formation of salt aggregates within the electrolyte film. As shown in Figure 3, lithium salt particles were observed to precipitate on the film surface, leading to increased light scattering and reduced optical transmission. These results indicate that transmittance decreases with increasing lithium salt concentration. To maintain a transmittance above 50%, LiClO_4_ concentrations of 0.1 or 0.2 M are preferred.

**Figure 2:**
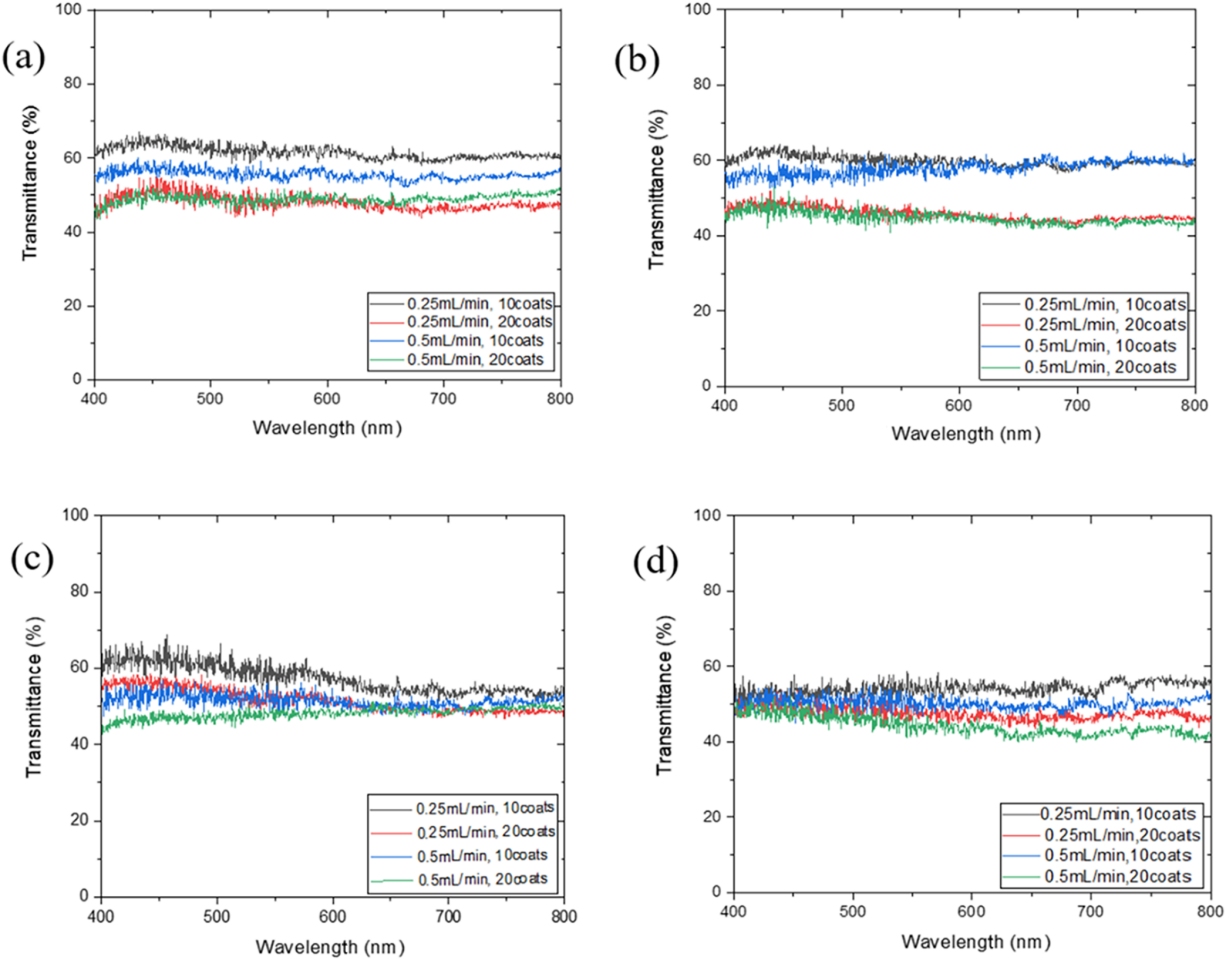
Transmittance of MMA/LiClO_4_ at different concentrations (a) 0.1 M, (b) 0.2 M, (c) 0.3 M, (d) 0.4 M.

**Figure 3:**
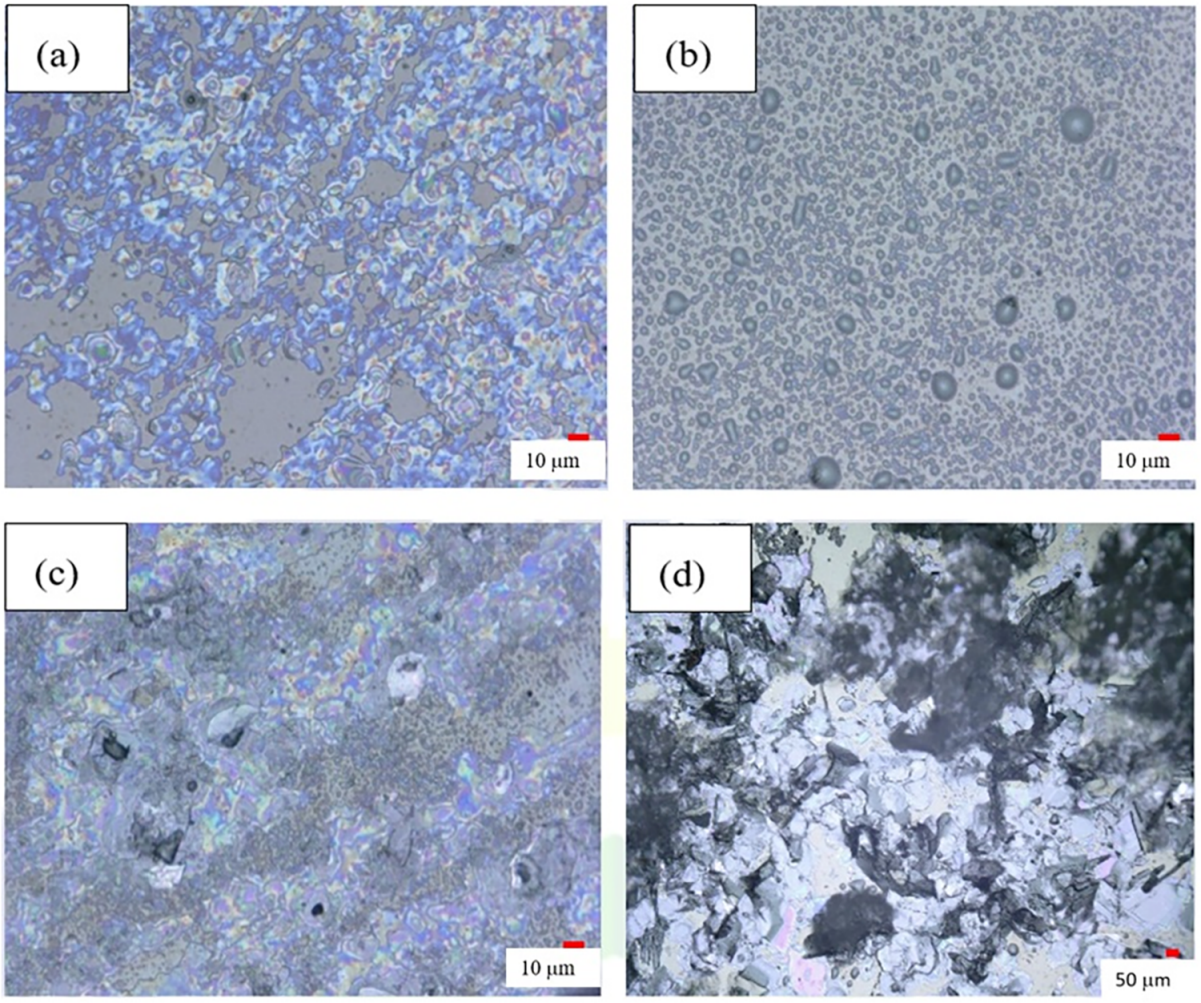
Optical microscopy of MMA/LiClO_4_ at different concentrations (a) 0.1 M, (b) 0.2 M, (c) 0.3 M, (d) 0.4 M.

**Table 1:** Optical Transmittance and Film Thickness of MMA/LiClO_4_ at Different Lithium Salt Concentration, Flow Rate, and Spray Coat

lithium salt concentration (M)	flow rate (ml min^–1^)	spray coat	optical transmittance (550 nm) (%)	film thickness (μm)
0.1	0.25	10	60	6.30
0.1	0.25	20	56	7.42
0.1	0.5	10	50	8.63
0.1	0.5	20	50	7.34
0.2	0.25	10	60	4.51
0.2	0.25	20	58	5.69
0.2	0.5	10	49	8.36
0.2	0.5	20	48	10.85
0.3	0.25	10	58	15.99
0.3	0.25	20	52	25.46
0.3	0.5	10	52	17.82
0.3	0.5	20	46	18.36
0.4	0.25	10	55	12.21
0.4	0.25	20	50	12.92
0.4	0.5	10	48	13.05
0.4	0.5	20	40	12.52

From Table 1, the optical transmittance and film thickness of the spray-coated polymer electrolyte films were strongly influenced by the lithium salt concentration, flow rate, and spray cycles. In general, increasing the salt concentration led to lower optical transparency and thicker films. This behavior may be attributed to enhanced light scattering, a higher refractive index mismatch, and an increased deposited mass.

Increasing the flow rate from 0.25 to 0.5 mL min^–1^ also reduced the transmittance from 56.1 to 47.9%, while slightly increasing the average film thickness from 11.88 to 12.87 μm. This indicates that higher precursor delivery promotes faster film buildup but compromises optical clarity.

Similarly, increasing the spray cycles from 10 to 20 coats increased the average thickness from 10.98 to 13.21 μm and decreased the average transmittance from 54.0 to 50.0%. These results confirm a clear trade-off between transparency and deposited film thickness.

Moreover, the obtained transmittance values are comparable to or better than those reported in previous studies (see Table 2).

**Table 2:** Transmittance Comparison of MMA/LiClO_4_ Electrolyte with Previous Reports

material	transmittance	references
MMA/LiClO_4_	60%	this work
gelatin/LiClO_4_	58%	[^60^]
PMMA/LiClO_4_	55%	[^61^]

The optical transmittance and film thickness of LiCl-based electrolyte films under different concentrations is presented in Figure 4 and Table 3. As shown in Figure 4a, the transmittance of 0.1 M LiCl ranged from 50 to 65%, demonstrating relatively high transparency. For the 0.2 M LiCl electrolyte (Figure 4b), the transmittance decreased slightly to 52–60%, indicating a minor influence of concentration. When the concentration was further increased to 0.3 M (Figure 4c), the transmittance decreased to 48–52%, suggesting a more pronounced concentration effect. At 0.4 M (Figure 4d), the transmittance decreased further with increasing salt concentration. Optical microscopy images (Figure 5) revealed that increasing lithium salt concentration led to the gradual precipitation of salt particles within the electrolyte films. This phenomenon reduced optical clarity and resulted in decreased transmittance, consistent with the observations for LiClO_4_-based systems. Overall, the results demonstrate that transmittance decreases with increasing lithium salt concentration. To maintain a transmittance above 50%, LiCl concentrations of 0.1–0.3 M are recommended. Notably, the 0.1 M LiCl electrolyte exhibited transmittance values exceeding 65%, indicating superior optical performance compared with previously reported systems (Table 4). The results clearly demonstrate that lithium salt concentration plays a critical role in determining the optical properties of polymer electrolyte films. At higher concentrations, salt aggregation and phase separation lead to increased light scattering, thereby reducing transmittance. In contrast, lower concentrations provide improved optical clarity while maintaining sufficient ionic transport properties. These findings suggest that electrolyte systems with optimized lithium salt concentrations (e.g., 0.1–0.2 M for LiClO_4_ and 0.1–0.3 M for LiCl) are suitable for applications requiring moderate transparency.

**Figure 4:**
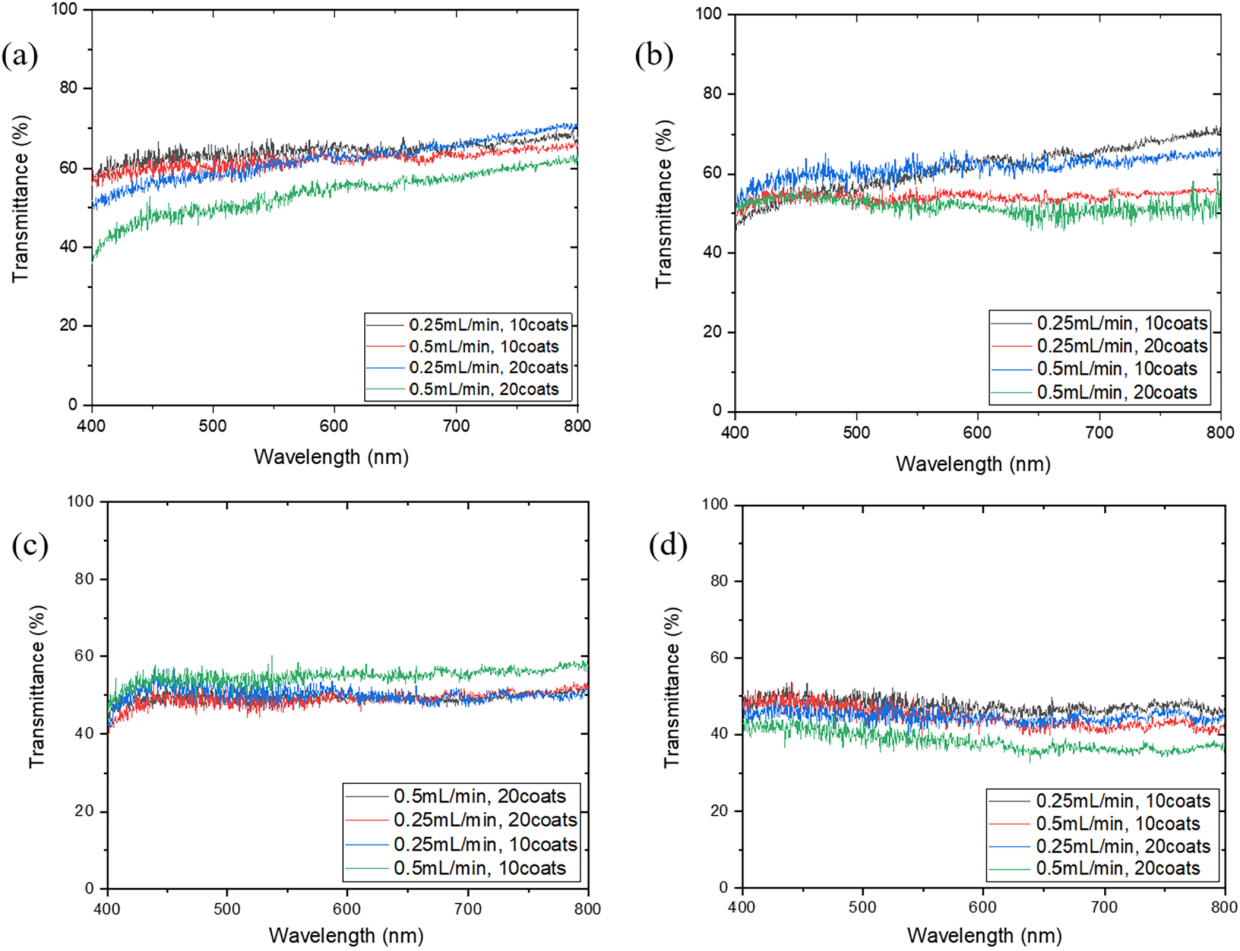
Transmittance of MMA/LiCl at different concentrations (a) 0.1 M, (b) 0.2 M, (c) 0.3 M, (d) 0.4 M.

**Figure 5:**
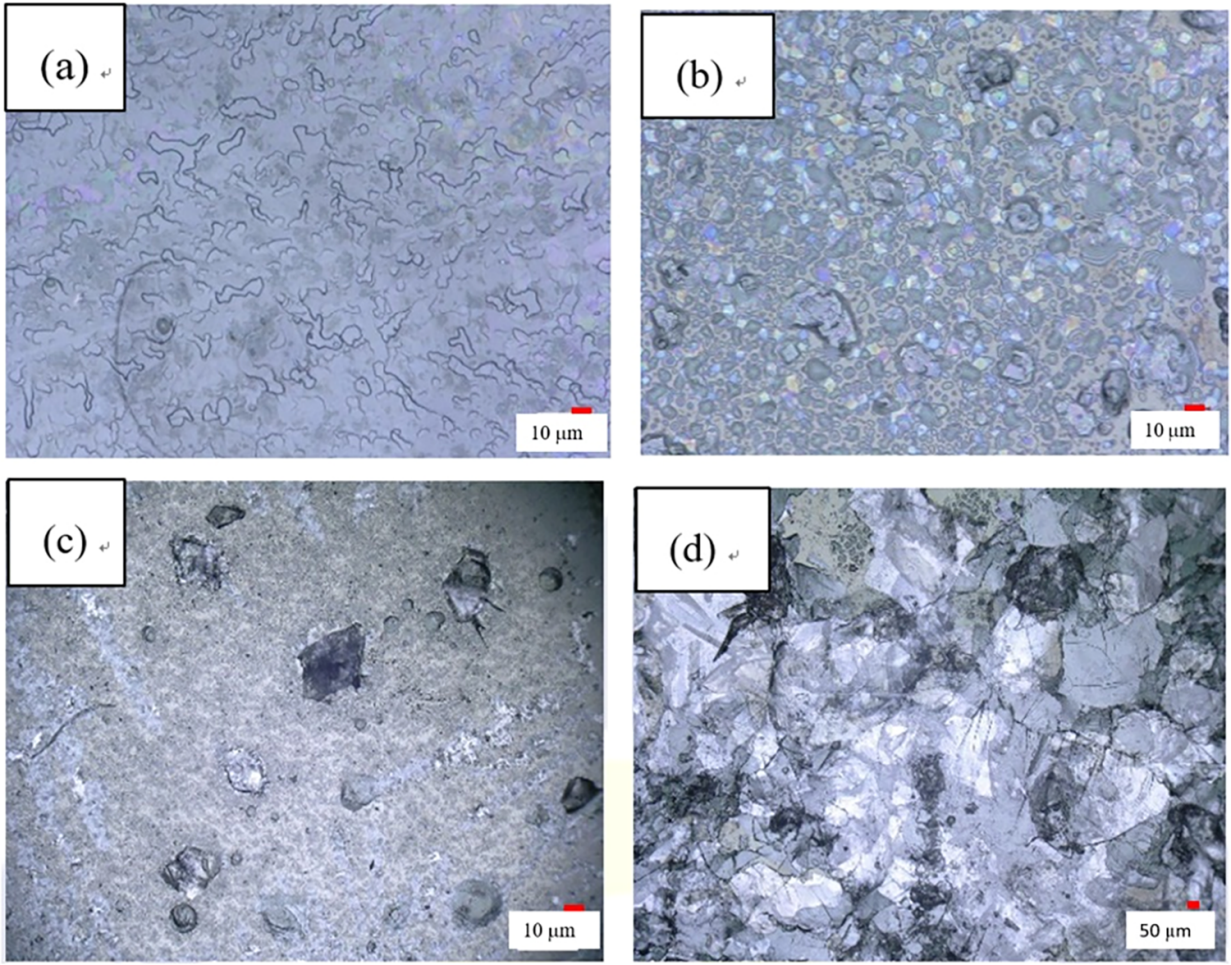
Optical microscopy of MMA/LiCl at different concentrations (a) 0.1 M, (b) 0.2 M, (c) 0.3 M, (d) 0.4 M.

**Table 3:** Optical Transmittance and Film Thickness of MMA/LiCl at Different Lithium Salt Concentration, Flow Rate and Spray Coat

lithium salt concentration (M)	flow rate (ml min^–1^)	spray coat	optical transmittance (550 nm) (%)	film thickness (μm)
0.1	0.25	10	65	2.81
0.1	0.25	20	60	5.47
0.1	0.5	10	60	3.71
0.1	0.5	20	50	13.31
0.2	0.25	10	58	2.79
0.2	0.25	20	60	5.95
0.2	0.5	10	55	3.53
0.2	0.5	20	52	8.64
0.3	0.25	10	52	3.50
0.3	0.25	20	50	4.79
0.3	0.5	10	48	5.35
0.3	0.5	20	52	9.85
0.4	0.25	10	50	13.05
0.4	0.25	20	48	13.02
0.4	0.5	10	48	13.31
0.4	0.5	20	39	13.07

**Table 4:** Transmittance Comparison of MMA/LiCl Electrolyte with Previous Reports

material	transmittance	references
MMA/LiCl	65%	this work
PEO/LiCl	65%	[^62^]
AAM/LiCl	60%	[^63^]
PVA/LiCl	60%	[^64^]

The optical transmittance and film thickness of the deposited electrolyte films were also strongly dependent on lithium salt concentration, flow rate, and spray coats. Increasing the salt concentration from 0.1 to 0.4 M reduced the average transmittance from 58.8 to 46.3%, while significantly increasing the film thickness from ∼6 to ∼13 μm. This behavior may be attributed to enhanced deposited mass, increased solution viscosity, and stronger light scattering.

Increasing the flow rate from 0.25 to 0.5 mL min^–1^ increased the average thickness from 6.32 to 8.09 μm, while reducing transmittance from 55.4 to 50.5%. Similarly, increasing the spray coats from 10 to 20 coats increased the thickness from 6.03 to 9.00 μm. These results also indicate a clear trade-off between optical transparency and film buildup. This phenomenon agrees with LiClO_4_ salt electrolyte in this study.

The primary objective of this work was to investigate the feasibility of ultrasonic spray deposition (USD) as a scalable, large-area, low-cost, and in-line fabrication technique for polymer electrolyte films, rather than to optimize the transparency of a complete electrochromic device. Accordingly, the present manuscript focuses on successful film deposition, process compatibility, ionic conductivity behavior, film formation quality, and potential applicability in electrochemical devices. Thus, the reported transmittance values should be viewed as an initial demonstration of optical suitability rather than the final optimized transparency performance. Although ultrahigh transparency is preferred for smart windows, transmittance values in the range of 50–65% can still be relevant for many practical applications, including privacy windows, glare-reduction coatings, indoor adaptive shading systems, display-related electrochromic layers, and laboratory prototypes or proof-of-concept devices. It also can be used in transparent supercapacitors, transparent thin-film energy-storage devices, integrated wearable/flexible electronics, multifunctional transparent electronics. Therefore, the moderate transparency remains meaningful depending on the intended application scenario.

The moderate transmittance may arise from several factors, including film thickness used for ionic conduction, surface scattering from solution-processed coatings, residual microstructural heterogeneity, and refractive index mismatch between the substrate and coating. These factors are common in early stage spray-coated electrolyte films and can be further improved through process optimization in future works.

Although optical microscopy does not provide nanoscale resolution, it remains a useful and widely accepted tool for evaluating film continuity, visible defects (cracks, pinholes, agglomeration), coating homogeneity, particle aggregation at the microscale, and large-area surface coverage. For solution-processed polymer electrolyte coatings, these features are directly relevant to the practical manufacturability and coating quality.

### 3.2 Ionic Conductivity

Electrochemical impedance spectroscopy (EIS) was employed to evaluate the ionic conductivity of LiClO_4_-based electrolyte films under various conditions. As shown in Figure 6 and Table 5, the ionic conductivity of the 0.1 M LiClO_4_ electrolyte, calculated from the measured resistance, film thickness, and electrode area, ranged from 1.829 × 10^–8^ to 2.886 × 10^–8^ S cm^–1^. For the 0.2 M LiClO_4_ electrolyte, the ionic conductivity increased to a range of 1.450 × 10^–8^ to 5.006 × 10^–8^ S cm^–1^. This enhancement indicates that a moderate increase in lithium salt concentration promotes ionic transport by improving ion dissociation and increasing the number of charge carriers. When the concentration was further increased to 0.3 M, the ionic conductivity was significantly enhanced, reaching values between 3.991 × 10^–8^ and 1.075 × 10^–7^ S cm^–1^. This improvement can be attributed to the increased availability of mobile lithium ions within the polymer matrix, which facilitates ion migration. However, for the 0.4 M LiClO_4_ electrolyte, a noticeable decrease in ionic conductivity was observed. This decline is attributed to the excessive precipitation of lithium salt within the electrolyte film, which leads to ion aggregation and obstructs ion transport pathways, thereby reducing ionic mobility.

**Figure 6:**
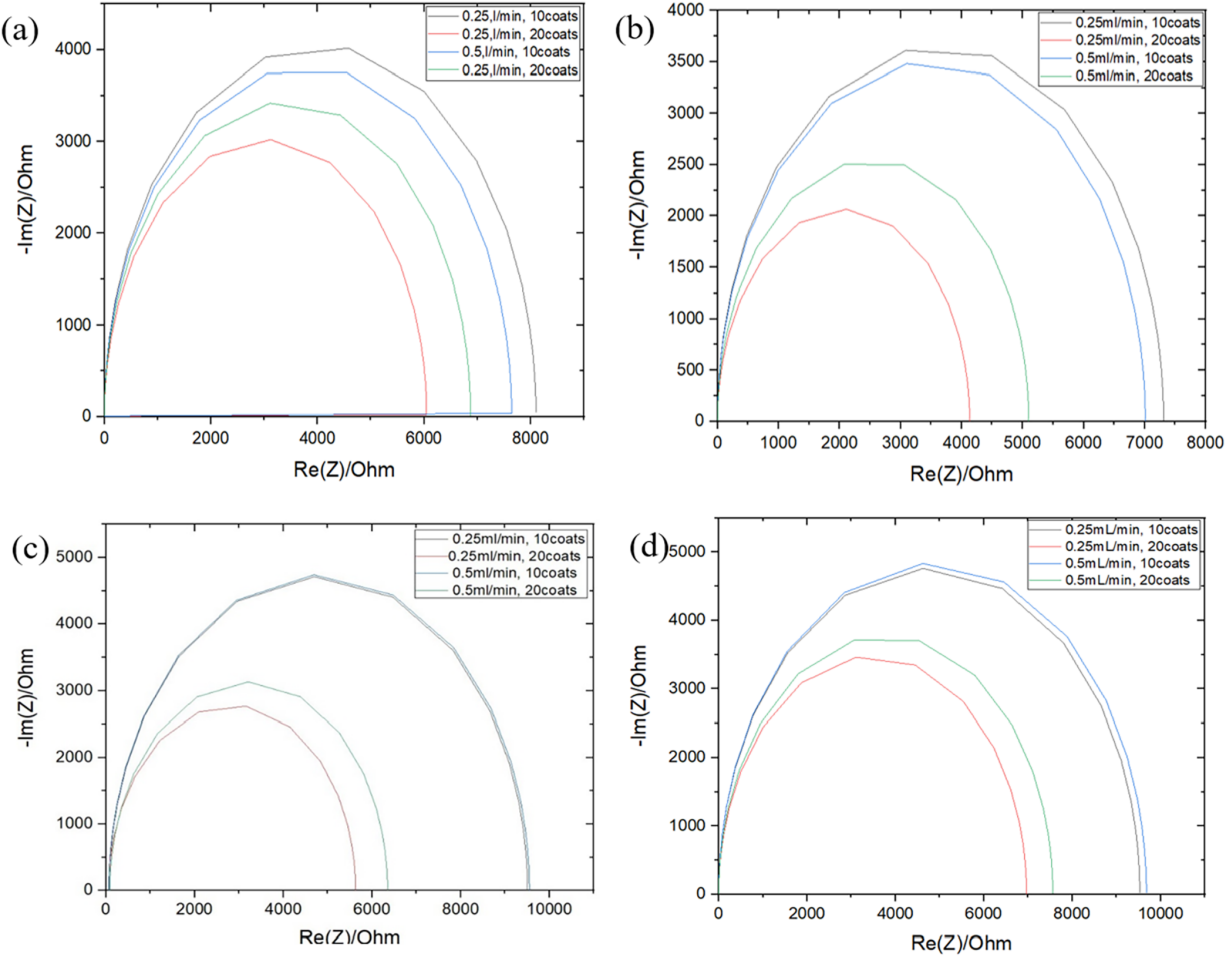
AC impedance diagram of LiClO_4_ at different concentrations. (a) 0.1 M, (b) 0.2 M, (c) 0.3 M, (d) 0.4 M.

**Table 5:** Optical Transmittance of MMA/LiClO_4_ at Different Lithium Salt Concentration, Flow Rate and Spray Coat

lithium salt concentration (M)	flow rate (ml min^–1^)	spray coat	ionic conductivity (S cm^–1^)	film thickness (μm)
0.1	0.25	10	1.829 × 10 ^–8^	6.30
0.1	0.25	20	2.655 × 10^–8^	7.42
0.1	0.5	10	2.886 × 10^–8^	8.63
0.1	0.5	20	2.513 × 10^–8^	7.34
0.2	0.25	10	1.450 × 10 ^–8^	4.51
0.2	0.25	20	2.806 × 10 ^–8^	5.69
0.2	0.5	10	3.239 × 10 ^–8^	8.36
0.2	0.5	20	5.006 × 10^–8^	10.85
0.3	0.25	10	3.991 × 10^–8^	15.99
0.3	0.25	20	4.421 × 10^–8^	25.46
0.3	0.5	10	1.075 × 10^–7^	17.82
0.3	0.5	20	6.899 × 10^–8^	18.36
0.4	0.25	10	3.024 × 10^–8^	12.21
0.4	0.25	20	4.323 × 10^–8^	12.92
0.4	0.5	10	3.181 × 10^–8^	13.05
0.4	0.5	20	3.876 × 10^–8^	12.52

Compared with previously reported polymer electrolyte systems (Table 6), the present electrolyte films exhibit improved ionic conductivity, particularly at higher LiClO_4_ concentrations. These results demonstrate that the combination of optimized lithium salt concentration and ultrasonic spray coating provides an effective strategy for fabricating polymer electrolyte films with an enhanced electrochemical performance.

**Table 6:** Ionic Conductivity Comparison of This Work (MMA/LiClO_4_) with Previous Reports

materials	ionic conductivity (S cm^–1^)	references
MMA/LiClO_4_	1.075 × 10^–7^	this work
MMA/PEG/LiClO_4_	8.78 × 10^–8^	[^59^]
MASTIFLEX/LiClO_4_	1.9 × 10^–8^	[^56^]

EIS measurements were also performed to assess the ionic transport properties of LiCl-based electrolyte films prepared under different conditions (Figure 7). As presented in Table 7, the ionic conductivity of the 0.1 M LiCl electrolyte was found to fall within the range of 4.461 × 10^–9^ to 2.147 × 10^–8^ S cm^–1^. For the 0.2 M LiCl system, the conductivity values decreased slightly, spanning from 3.856 × 10^–9^ to 1.341 × 10^–8^ S cm^–1^. In the case of the 0.3 M LiCl electrolyte, the conductivity ranged from 5.089 × 10^–9^ to 1.110 × 10^–8^ S cm^–1^.

**Figure 7:**
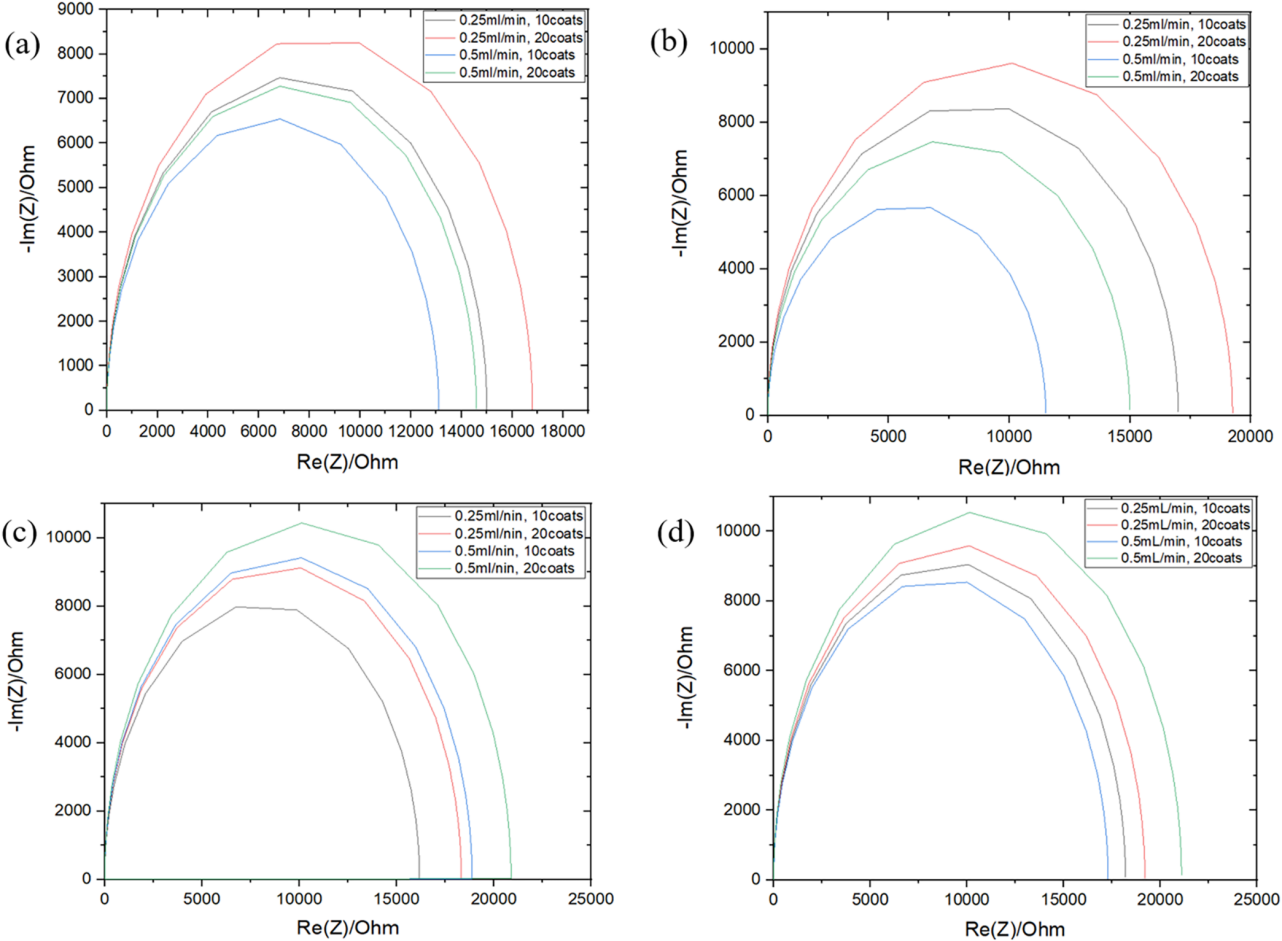
AC impedance diagram of LiCl at different concentrations. (a) 0.1 M, (b) 0.2 M, (c) 0.3 M, (d) 0.4 M.

**Table 7:** Optical Transmittance of MMA/LiCl at Different Lithium Salt Concentration, Flow Rate and Spray Coat

lithium salt concentration (M)	flow rate (ml min^–1^)	spray coat	ionic conductivity (S cm^–1^)	film thickness (μm)
0.1	0.25	10	4.461 × 10^–9^	2.81
0.1	0.25	20	6.664 × 10^–9^	5.47
0.1	0.5	10	7.657 × 10^–9^	3.71
0.1	0.5	20	2.147 × 10^–8^	13.31
0.2	0.25	10	3.856 × 10^–9^	2.79
0.2	0.25	20	7.209 × 10^–9^	5.95
0.2	0.5	10	7.171 × 10^–9^	3.53
0.2	0.5	20	1.341 × 10^–8^	8.64
0.3	0.25	10	5.089 × 10^–8^	3.50
0.3	0.25	20	6.660 × 10^–8^	4.79
0.3	0.5	10	6.151 × 10^–7^	5.35
0.3	0.5	20	1.110 × 10^–8^	9.85
0.4	0.25	10	1.681 × 10^–8^	13.05
0.4	0.25	20	1.593 × 10^–8^	13.02
0.4	0.5	10	1.786 × 10^–8^	13.31
0.4	0.5	20	1.447 × 10^–8^	13.07

A general trend of increasing ionic conductivity with a higher number of spray-coating layers was observed. This behavior suggests that thicker and more continuous electrolyte films improve ion migration by reducing the interfacial resistance and enhancing transport pathways. The highest conductivity values were consistently achieved at higher coating layer numbers.

When the lithium salt concentration was increased to 0.4 M, a decrease in ionic conductivity was observed. This decrease is likely associated with the formation of salt-rich domains within the polymer matrix, which promotes ion aggregation and hinders effective ion diffusion. As a result, the overall ionic mobility is suppressed at high salt concentrations.

Tables 5 and 7 consistently demonstrated that the ionic conductivity strongly depended on lithium salt concentration and spray parameters. In both cases, the highest conductivity was obtained at 0.3 M lithium salt concentration under a flow rate of 0.5 mL min^–1^ and 10 spray coats, indicating the existence of an optimum salt content for ion transport.

Below this concentration, the number of mobile ions was insufficient, whereas excessive salt loading likely induced ion pairing and reduced polymer chain mobility. The film thickness increased with increasing coating cycles and, in some cases, with higher salt concentration. However, thicker films did not necessarily exhibit higher conductivity, confirming that ionic transport was governed mainly by the internal electrolyte structure rather than by thickness alone. These results demonstrate that ultrasonic spray deposition provides an effective route for tuning both the film geometry and electrochemical performance through process control.

The ion transport behavior can be reasonably interpreted on the basis of established polymer electrolyte theory together with the conductivity trends observed in this work. The ionic conductivity of polymer electrolytes is generally determined by two key factors: concentration of free mobile ions, generated through dissolution and dissociation of the lithium salt. And ion mobility depends on local polymer chain motion and free volume within the polymer matrix. After salt incorporation, Li^+^ ions coordinate with polar functional groups of the polymer host, and ionic migration occurs through hopping between transient coordination sites, assisted by segmental motion of the polymer chains.

At low salt concentration, the number of available charge carriers is limited, resulting in low conductivity. As salt concentration increases, more dissociated ions become available, leading to improved conductivity. However, at excessive salt loading, conductivity may no longer increase due to ion pairing or ion aggregation, stronger ion–polymer interactions, reduced chain flexibility, and lower free volume. These competing effects explain the nonlinear conductivity trend observed in the experimental results. In solid polymer electrolytes, ion transport is strongly coupled with polymer segmental relaxation. Increased chain flexibility facilitates transient pathways for ion hopping, whereas rigid or highly ordered polymer domains hinder ion movement. Therefore, conductivity enhancement is not solely controlled by salt concentration, but also by the mobility of the polymer matrix.

Successful UV curing was confirmed through the observed macroscopic properties of the films, including formation of self-supporting solid films, nonflowing and mechanically stable coatings after exposure, strong adhesion to the substrate, no visible dissolution or deformation during handling, and reproducible electrochemical measurements after curing. These results indicate that sufficient polymerization/cross-linking occurred to generate a stable solid electrolyte network. For the purpose of this paper, the key requirement was to demonstrate that UV curing can be effectively integrated with the USD process to produce functional polymer electrolyte films suitable for electrochemical characterization. From an application perspective, the successful transition from liquid precursor to solid, continuous, and operational electrolyte films is the most relevant outcome of the curing process in this initial study.

Future work will include advanced structural characterization (e.g., FTIR, XRD, and DSC) to further validate the proposed transport mechanism.

Compared with previously reported polymer electrolyte systems, as shown in Table 8, the electrolyte films developed in this work demonstrate a relatively higher ionic conductivity. This improvement highlights the capability of ultrasonic spray coating as a reliable method for producing polymer electrolyte films with an enhanced electrochemical performance. These results indicate that optimizing both the salt concentration and film morphology is essential to achieve higher ionic conductivity in polymer electrolyte systems.

**Table 8:** Ionic Conductivity Comparison of This Work (MMA/LiClO_4_) with Previous Reports

materials	ionic conductivity (S cm^–1^)	references
MMA/LiCl	6.151 × 10^–7^	this work
MMA/PEG/LiClO_4_	1.0 × 10^–8^	[^59^]
PEO/LiCl	2.0 × 10^–8^	[^65^]

The conductivity value of 6.151 × 10^–7^ S cm^–1^ at 0.3 M LiCl at this concentration is attributed to a local optimum in salt dissociation and temporary enhancement of amorphous polymer chain mobility, which can occasionally occur near a critical salt concentration. At lower salt contents, the number of charge carriers is insufficient, whereas at higher salt contents, ion pairing/aggregation and reduced segmental motion may suppress ionic transport.

Typical high-conductivity PMMA-based GPEs usually contain large amounts of liquid solvents or plasticizers, swollen polymer networks, high free volume, and liquid-assisted ion transport pathways. Under these conditions, ion migration occurs partly through liquid-like domains, which greatly enhances ionic conductivity. In contrast, the present system is closer to a solid/quasi-solid UV-cured polymer electrolyte film, where ionic transport mainly depends on salt dissociation, hopping of ions between coordination sites, and local polymer segmental motion. This mechanism is inherently slower than that of solvent-rich gel systems.

After UV curing, the polymer matrix forms a stable network structure that improves film integrity, dimensional stability, mechanical robustness, and leakage resistance. However, this cross-linked structure can also restrict polymer chain mobility and reduce free volume, thereby lowering ion transport efficiency compared with flexible gel electrolytes. Thus, there is a common trade-off between the conductivity and structural stability.

Many literature PMMA-GPE systems achieve high conductivity because they incorporate substantial amounts of propylene carbonate (PC), ethylene carbonate (EC), ionic liquids, and low-molecular-weight plasticizers. These components facilitate the ion dissociation and mobility. The present work did not aim to maximize conductivity through high solvent loading but instead focused on developing process-compatible solid films fabricated by ultrasonic spray deposition (USD).

Without large amounts of plasticizer, the segmental motion of the polymer chains is more restricted, reducing ion mobility. The lower ionic conductivity can also be due to incomplete salt dissociation and possible ion pairing/aggregation decreasing the concentration of mobile charge carriers. Spray-deposited solid films may possess a lower solvent content and more compact morphology than swollen gels, limiting ionic transport pathways.

The present work focused on feasibility demonstration rather than full compositional optimization (additives, copolymers, fillers, post-treatment, etc.). Although the conductivity is lower than that of highly optimized GPEs, the results remain scientifically meaningful because they demonstrate that polymer electrolyte films can be continuously fabricated by USD, conductivity is tunable through salt selection and concentration, functional electrolyte coatings are achievable, and the process is promising for scalable electrochemical device manufacturing. These findings provide an important platform for future optimizations.

To better the ionic conductivity, future research will include further improvement of plasticizer incorporation, ionic liquid addition, polymer blending, nanofiller introduction, reduced crystallinity, and postannealing treatment.

The main novelty of this paper lies in demonstrating that USD combined with UV curing can produce scalable large-area electrolyte coatings, uniform thin films, low-cost and in-line processable electrolytes, and mechanically stable solid-state electrolyte layers.

Therefore, the present paper was intentionally focused on the materials/processing aspect rather than full device integration at this stage. This work serves as a foundational study demonstrating that uniform polymer electrolyte films can be successfully fabricated by the USD technique. Establishing a reliable deposition route is a necessary first step before integration into practical electrochemical devices.

Compared with conventional casting methods, USD offers several practical advantages, including scalable large-area coating, compatibility with roll-to-roll/in-line manufacturing, reduced material waste, controllable film thickness, and potential integration with flexible devices. These processing advantages are highly relevant for future industrial applications. The device-level studies, including electrochromic switching performance, solid-state supercapacitor testing, battery cell evaluation, and long-term cycling stability, are currently under investigation and will be reported in future work.

Ultrasonic spray deposition (USD) generates fine droplets that are deposited in a controlled layer-by-layer manner, enabling more uniform coating coverage, better thickness control, reduced local thickness variation, and fewer macroscopic defects. A more homogeneous electrolyte layer can reduce tortuous ion pathways and improve the reproducibility of the ionic transport across the film. In contrast, conventional casting often produces thicker films with less precise thickness control and possible inhomogeneity during the solvent evaporation.

During USD, micrometer-sized droplets rapidly spread and dry on the substrate surface. This can lead to more uniform solvent evaporation, reduced phase separation, less large-scale salt segregation, and better distribution of ionic species within the film. By comparison, slow solvent evaporation in cast films may sometimes promote concentration gradients or localized aggregation, which can hinder the ion transport.

USD is particularly suitable for fabricating thin electrolyte coatings. A shorter transport distance through a thinner film can lower the effective ionic resistance of the device, even if the intrinsic conductivity remains similar. Thus, from a device perspective, spray-coated thin films may exhibit a better electrochemical response than thicker cast membranes. Spray coating is highly compatible with sequential layer deposition and direct coating onto electrodes and substrates. Improved interfacial contact between the electrolyte and electrode can reduce interfacial resistance and facilitate ion transfer at the electrode/electrolyte boundary. This is especially important for practical electrochemical devices.

We also emphasize that USD does not automatically guarantee higher intrinsic conductivity than casting. The final ionic conductivity still depends on polymer chemistry, salt concentration, plasticizer content, curing conditions, and microstructure. The main benefit of USD is therefore process-enabled structural optimization and scalable thin-film fabrication rather than a direct change in transport physics.

GPEs have attracted considerable attention in energy-storage applications because they combine the high ionic conductivity of liquid electrolytes with the improved safety and mechanical stability of solid polymer matrices.^66–68^ Compared with conventional liquid electrolytes, GPEs can reduce leakage risk, improve dimensional integrity, and offer better compatibility with flexible and thin-film devices.^67,68^

In particular, Seol et al. systematically compared five printable GPE formulations for solid-state printed supercapacitors and demonstrated that electrolyte composition strongly affects ionic conductivity, printability, electrochemical response, and device performance.^66^ Their work highlighted that practical electrolyte development should not focus solely on conductivity but also on processability and scalable manufacturing. This concept is highly relevant to the present study, where ultrasonic spray deposition (USD) is explored as an alternative scalable coating technique for polymer electrolyte fabrication.

For rechargeable batteries, GPEs are widely studied because they provide safer operation than volatile liquid electrolytes while maintaining acceptable ionic conductivity. PMMA-based and PVDF-HFP-based GPEs have been reported to exhibit ionic conductivities in the range of 10^–4^–10^–3^ S cm^–1^ when combined with carbonate solvents or plasticizers.^67,69,70^ Such high conductivity is generally attributed to enhanced salt dissociation, increased free volume, and improved polymer chain mobility within swollen gel networks.^68,70^

For supercapacitor applications, GPEs are also highly attractive due to their ability to function simultaneously as separator and electrolyte while enabling flexible or printed device architectures.^66,71^ Previous studies have shown that GPE composition strongly influences equivalent series resistance (ESR), capacitance retention, rate capability, and cycling stability.^66,71,72^ Therefore, the optimization of electrolyte formulation is essential for maximizing device performance.

In addition to batteries and supercapacitors, GPEs are increasingly relevant for transparent and multifunctional electrochemical systems, including electrochromic devices, smart windows, and wearable electronics.^73,74^ In such applications, film transparency, mechanical flexibility, and scalable coating processes become important alongside the ionic transport performance.

Although the ionic conductivity of the present UV-cured spray-coated electrolyte is lower than that of highly plasticized GPE systems, this result is understandable because the current formulation behaves more like a solid or quasi-solid network, where ion transport mainly relies on ion hopping assisted by local polymer segmental motion rather than liquid-like diffusion pathways.^67,68^ Nevertheless, the present system offers several advantages, including compatibility with ultrasonic spray coating, uniform thin-film deposition, potential for roll-to-roll or in-line production, transparent coating capability, reduced material waste, and improved dimensional stability after UV curing.

Previous literature has shown that conductivity in such systems can be further improved through strategies such as plasticizer optimization, ionic liquid incorporation, nanofiller addition (Al_2_O_3_, SiO_2_), polymer blending, and salt concentration tuning.^70,75–77^ Therefore, the present study provides a useful processing platform for future development of high-performance spray-coated GPEs for next-generation energy-storage devices.

While previous studies such as Seol et al. emphasized printable GPEs for supercapacitors,^66^ the present work complements the field by demonstrating that ultrasonic spray deposition is another promising scalable route for manufacturing functional polymer electrolyte films for advanced energy-storage applications. The future work will include more electrochemical validation, including CV, galvanostatic charge–discharge, electrochemical stability window, cycling stability, and device-level testing.

### 3.3 Mechanical Properties Test

The mechanical robustness of polymer electrolyte films is essential to ensure their structural stability under external stress or pressure, thereby preventing mechanical failure, such as cracking, delamination, or device malfunction.

The hardness and elastic modulus of LiClO_4_-based electrolyte films at different concentrations are summarized in Figures 8 and 9. At a concentration of 0.1 M, the electrolyte exhibited a relatively low hardness of 0.04 GPa and an elastic modulus of 1.4 GPa. When the concentration was increased to 0.2 M, both mechanical properties were significantly enhanced, with hardness reaching 1.2 GPa and the elastic modulus increasing to 2.8 GPa.

**Figure 8:**
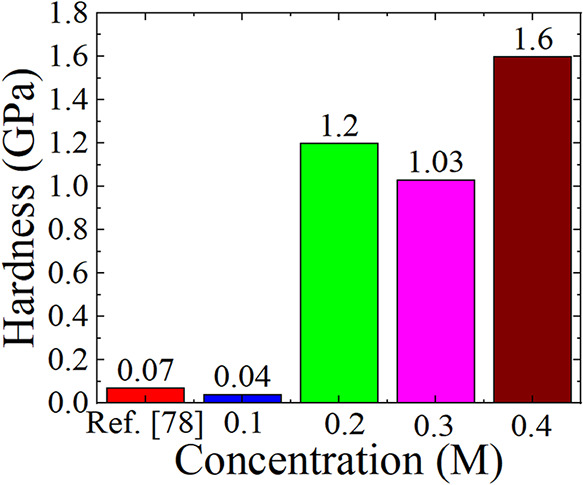
Hardness of MMA/LiClO_4_ at different concentration and previous report.

**Figure 9:**
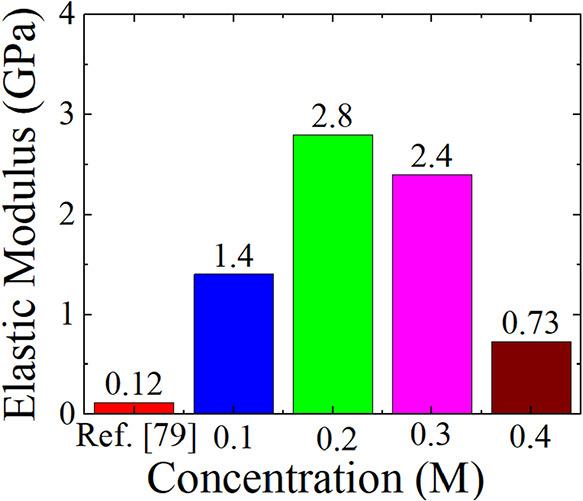
Elastic modulus of MMA/LiClO_4_ at different concentration and previous report.

At 0.3 M, the hardness slightly decreased to 1.03 GPa, accompanied by a substantial reduction in the elastic modulus to 2.4 GPa. When the concentration was further increased to 0.4 M, the hardness increased again to 1.6 GPa, while the elastic modulus remained relatively low at 0.73 GPa.

Compared with previously reported values (hardness: 0.07 GPa,^78^ elastic modulus: 0.12 GPa^79^), the present electrolyte films exhibit significantly improved mechanical strength. The enhanced hardness indicates improved resistance to external deformation, while variations in elastic modulus suggest that lithium salt concentration strongly influences the internal structure and mechanical integrity of the polymer matrix.

These results demonstrate that optimizing the lithium salt concentration is critical for achieving a balance between mechanical strength and structural stability in polymer electrolyte systems.

The mechanical properties of LiCl-based electrolyte films were evaluated in terms of hardness and elastic modulus at different lithium salt concentrations, as shown in Figures 10 and 11.

**Figure 10:**
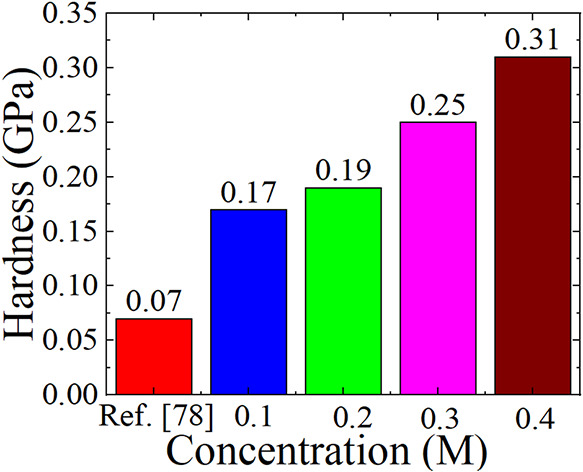
Hardness of MMA/LiCl at different concentration and previous report.

**Figure 11:**
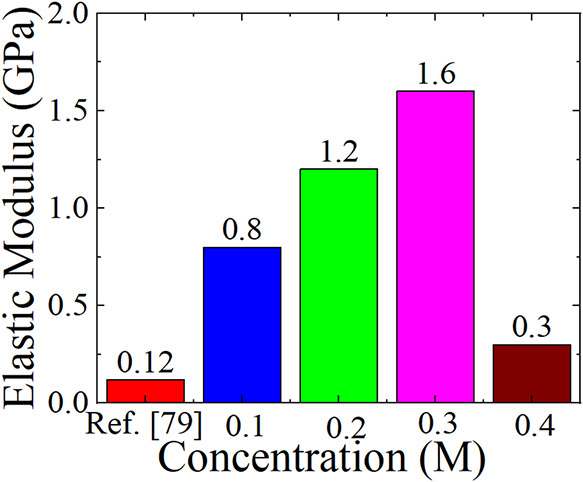
Elastic modulus of MMA/LiCl at different concentration and previous report.

At a concentration of 0.1 M, the electrolyte film exhibited a hardness of 0.17 GPa and an elastic modulus of 0.8 GPa, indicating a moderate mechanical strength. When the concentration was increased to 0.2 M, both hardness and elastic modulus were increased to 0.19 and 1.2 GPa, respectively, suggesting an increase in the structural rigidity.

At 0.3 M, the hardness increased to 0.25 GPa, accompanied by a significant increase in the elastic modulus to 1.6 GPa. This enhancement may be attributed to improved intermolecular interactions or localized densification within the polymer matrix at this concentration. However, when the concentration was further increased to 0.4 M, both hardness was increased to 0.31 GPa, but elastic modulus was decreased sharply to 0.3 GPa, respectively. This decline is likely due to excessive lithium salt incorporation, leading to structural inhomogeneity and a reduced mechanical integrity.

Compared with previously reported values (hardness: 0.07 GPa,^78^ elastic modulus: 0.12 GPa^79^), the electrolyte films prepared in this study exhibit improved mechanical performance under optimized conditions. These results indicate that lithium salt concentration plays a crucial role in determining the mechanical stability of polymer electrolyte systems.

Hardness was included in this study as a mechanical property indicator to evaluate the physical robustness of UV-cured polymer electrolyte films. Since the present work focuses on ultrasonic spray deposition (USD) and UV curing of thin polymer electrolyte coatings, mechanical integrity is an important practical consideration for film handling, coating durability, and device fabrication compatibility.

For thin-film polymer electrolytes, insufficient mechanical strength may lead to surface damage during handling, scratching during assembly, deformation during lamination, reduced dimensional stability, and poor long-term reliability. Therefore, hardness testing was used as a simple and practical method to assess whether the cured films possessed adequate structural stability after processing.

Although hardness may be less relevant for conventional bulk battery electrolytes, it becomes more meaningful for coated solid-state electrolyte layers, thin-film electrochromic devices, flexible multilayer devices, roll-to-roll-processed coatings, and transparent functional films requiring surface durability. In these applications, mechanical surface robustness can influence the manufacturability and operational lifetime.

## 4 Conclusions

In this study, two types of lithium-salt-based gel polymer electrolytes were successfully fabricated using a simple and scalable ultrasonic spray-coating technique. The effects of the lithium salt concentration, spray flow rate, and number of coating layers on the optical and electrochemical properties were systematically investigated. Variations in the optical transmittance and ionic conductivity were clearly observed as a function of these processing parameters.

The results demonstrate that the electrolyte films exhibit favorable optical and electrochemical performance. The optical transmittance reached up to 60% for LiClO_4_-based systems and 65% for LiCl-based systems. In terms of ionic conductivity, LiClO_4_ exhibited superior performance with a maximum value of 1.075 × 10^–7^ S cm^–1^, while LiCl reached 6.151 × 10^–7^ S cm^–1^. Additionally, the electrolyte films showed excellent mechanical properties, with hardness values exceeding 1.6 GPa and elastic moduli above 2.8 GPa, indicating sufficient mechanical robustness for practical applications.

Overall, the ultrasonic spray-coating method provides a high material utilization rate, uniform film morphology, and scalability for large-area in-line fabrication. The developed electrolyte systems exhibit a promising balance among optical transparency, ionic conductivity, and mechanical stability. These characteristics make them suitable candidates for applications in electrochromic devices and other energy-related systems.
